# Influencing factors on missed opportunities for vaccination among children aged 0-24 months in hospitals of White Nile State, Sudan: a mixed method study

**DOI:** 10.11604/pamj.2025.52.43.48136

**Published:** 2025-09-29

**Authors:** Abu Obeida Babiker Ahmed Eltayeb, Ariya Bunngamchairat, Kwanjai Amnatsatsue, Jutatip Sillabutra, Kanittha Chamroonsawasdi, Dale Allan Rhoda

**Affiliations:** 1 Faculty of Public Health, Mahidol University, Bangkok, Thailand,; 2 Biostat Global Consulting, Worthington, OH 43085, USA

**Keywords:** Immunisation, missed opportunities for vaccination, White Nile State

## Abstract

**Introduction:**

Missed Opportunities for Vaccination (MOV) is one of the reasons contributing to low and inequitable vaccination coverage. We conducted a mixed method study to assess prevalence of MOVs among children aged 0-24 months in hospitals of White Nile State, Sudan during 2023 as well as to identify underlying causes of MOVs.

**Methods:**

for quantitative assessment, caregivers of 226 children with vaccination cards and ages 0-24 months (mean 8.3 months; standard deviation 5.7; 45% male) were interviewed when they visited 18 hospitals. Stata v18 and the programme Missed Opportunities Vaccination Coverage Quality Indicators (MISS VCQI) were used to analyse the children's vaccination history data. Multivariable logistic regression was used to assess association between the main outcome variable (occurrence of MOVs) and independent variables. For a qualitative perspective, hospital documents and Ministry of Health policies were reviewed for MOV-related content. Ideas about underlying causes of MOVs were elicited in focus group discussions with health workers and caregivers and in in-depth interviews with hospital managers and senior staff.

**Results:**

on the study day, 187 children were eligible for one or more doses. Fifty-seven (30.5%) received all the doses they were due and 130 (69.5%) experienced MOVs. Of the 678 doses that were due, 445 (65.6%) were received and 233 (34.3%) were not. MOVs were most prevalent for the dose BCG (63.2%) and least prevalent for OPV (6.5%). Broadening the analysis to all 226 children with cards and to all their documented vaccination visits both on and before the study day, 152 (67.3%) had experienced an MOV on at least one vaccination visit. MOVs were less likely to occur in a) children who were born at a health facility (aOR 0.52, 95% CI 0.29-0.95; P=.03), b) children whose caregiver has secondary or higher education (aOR 0.35, 95% CI 0.18-0.67; P=0.002), and c) in families where the father alone makes decisions about childhood vaccination (aOR 0.31, 95% CI 0.14-0.69; P=0.004). Stakeholders identified likely reasons for MOVs, including stock deficiencies of vaccines and syringes, inconvenient scheduling of vaccination sessions, and health worker aversion to opening a multi-dose vaccine vial for a single child. We found no federal or state level policies in place to address MOVs.

**Conclusion:**

quantitatively, a high prevalence of MOVs was observed which contributes to low vaccination coverage in White Nile State. Qualitatively, stakeholders identified factors that might be addressed to minimize the occurrence of MOVs.

## Introduction

The World Health Organization's Immunisation Agenda 2030 (IA2030) advocates for equitable vaccine access and urges countries to create national plans to address immunisation inequities. By 2030, it aims to halve the number of zero-dose children, introduce new vaccines, and achieve 90% immunisation coverage [1]. The government of Sudan aims for universal health coverage by providing primary health care services, including immunisation, across the country [2,3]. However, immunisation coverage has declined due to a weak health system, humanitarian crises, security issues, and the COVID-19 pandemic. WHO and UNICEF reported a drop in DTP3 coverage from 93% in 2019 to 51% in 2023 [4]. Reasons for under-vaccination are in general related to supply and demand factors, and they vary by country, region, and population segment.

The WHO defines a missed opportunity for vaccination (MOV) as any health service contact where an eligible individual does not receive all vaccines that are due [5]. Health systems should use every contact with a patient to check vaccination status and offer missing doses, especially for children at risk of vaccine-preventable diseases [6]. On an occasion when a patient is vaccinated with some but not all of the doses for which they are eligible, it is called missed opportunity for simultaneous vaccination (MOSV). The term MOV is used to refer to either a MOSV or an occasion where the patient is eligible and does not receive any doses. So MOSVs are subset of the broader term MOV [7]. MOVs contribute to low and inequitable vaccination coverage [8]. Reducing their prevalence can enhance immunisation coverage, timeliness of vaccination, and overall health service delivery and can promote synergy between treatment services and preventive programmes which may result in additional patient care benefits beyond immunisation [5]. A 2014 review of 59 studies across 26 countries and 23 years found that MOV prevalence at 32.2% among children and 46.9% among women of child-bearing age, with rates varying by region [9]. A 2025 meta-analysis of nationally representative DHS and MICS surveys in sub-Saharan Africa found a pooled prevalence of 34% of children ages 12-23 months experienced one or more MOVs, with rates that range from 11-97% [10].

Sudan's health facilities serve many patients daily. In light of the IA2030 goals, it is relevant to assess whether workers at those facilities identify and vaccinate eligible children at every opportunity [11]. In Sudan, particularly White Nile State, no recent studies have documented MOV prevalence. Evidence is needed to understand the prevalence and causes of MOVs [12]. This study aimed to a) estimate the prevalence of MOVs among children ages 0-24 months in hospitals of White Nile State, b) to assess factors associated with MOVs, c) document stakeholders' perceptions of underlying causes of MOVs in those hospitals, d) search government documents and policies for guidance about minimising MOVs.

## Methods

**Study design and setting:** this manuscript describes a mixed method study with concurrent quantitative and qualitative arms [13]. The qualitative information was used to help interpret the quantitative work and provide insight on analysis outcomes [14,15]. The study followed steps recently updated by WHO [5,16]. White Nile State comprises 9 administrative localities that hold altogether 30 hospitals that offer immunisation services free of charge. Due to resource limitations the study was conducted in 18 hospitals.

**Study population and sampling:** all three private hospitals were included in the sample by design and 15 of 27 public hospitals were selected using a stratified random sample with localities serving as strata. At each selected hospital, a quota sampling method was used, conducting exit interviews with parents of children with vaccination cards. MOV exit interview studies use purposive sampling of health facilities and use convenience sampling at the study site and so do not purport to obtain a rigorously random probability sample [15]. The goal is to speak with enough caregivers from enough places to obtain a robust sample of outcomes and experiences. Our goal at each selected hospital was to conduct 10-11 interviews with caregivers of children who had immunisation cards with some immunisation dates written on them. Two types of focus group discussions (FGDs) were conducted in the hospitals: one with mothers/caregivers and one with healthcare workers. Children whose caregivers participated in the FGDs were not included in the quantitative sample to reduce bias [17]. Each caregiver FGD was meant to include three caregivers of children ages 0-11 months and three of children 12-24 months. Every health worker present on the interview day was included in the health worker FGDs, regardless of their involvement in immunisation services [18]. In-depth interviews (IDIs) were conducted with one-to-two staff persons from each hospital in addition to immunisation managers from federal and state Ministry of Health, primary healthcare directorate and locality health office. A total of 30 IDIs and 14 FGDs were held.

**Data collection:** the quantitative arm involved exit interviews with parents/caregivers who were accompanying children aged 0-24 months to the hospital. If the caregiver was in possession of the child's immunisation card, the study interviewer transcribed all the vaccination dates from the card. The caregiver was asked whether the child had received any vaccinations during today's visit to the hospital and if not, why not. In subsequent analysis, dates from the card were used to evaluate whether the child had been eligible for any doses on that day and whether they had experienced any MOVs. The qualitative arm aimed to understand why vaccination opportunities are missed in hospitals. It included focus group discussions (FGDs) with parents/caregivers and (separately) with health workers as well as in-depth interviews (IDIs) with hospital directors and health administrators, and interviews with health officials, a health workers knowledge and practices (KAP) questionnaire, and a review of relevant policy documents. Data from both arms were collected from August through December 2023.

**Statistical analysis:** all quantitative data were analysed using Stata v18 [19]. Special permission was obtained to use the Missed Opportunities Vaccination Coverage Quality Indicators (MISS VCQI) suite of programmes that was developed for the Pan American Health Organization by Biostat Global Consulting. The analysis identified children with documented vaccination dates, those due for at least one dose on the study day (OSD), and those with at least one MOV or MOSV. Similarly, the software examined all the earlier dates when each child had received vaccine doses and tallied whether they received a) any doses they were not eligible for, b) any doses they were eligible for, and c) all of the doses they were eligible for. The MISS VCQI software allows estimation of prevalence of MOVs on the study day itself (which we abbreviate OSD) and prevalence of MOSVs (only) on all of the children's vaccination dates before the study day (BSD). Note that the software is not aware of all the dates when the child had contact with the health system before the study day; it only knows the list of dates when the child received one or more vaccines. So, if the child had some occasions BSD when they were eligible, but not vaccinated at all, the analysis cannot identify those.

To assess correlation between demographic categorical variables and whether a child had experienced one or more MOVs (either OSD or BSD), three steps were followed. First, cross-tabulation identified the percentage of children in each demographic category with one or more MOVs. Second, univariate survey-adjusted logistic regression estimated the association between the outcome and each variable, treating each facility as its own survey stratum. Third, variables with a univariate p-value smaller than 0.20 were included as candidates in a multivariable logistic regression model. Candidate variables with a multivariate p-value higher than 0.05 were dropped iteratively, starting with the one with the highest p-value, until all remaining variables had a p-value smaller than 0.05. Finally, each dropped variable was re-introduced into the model to assess whether it changed regression coefficients by 20% or more. If yes, it was retained. The final model identified significant associations after adjusting for other variables. Model fit was assessed with the Archer-Lemeshow goodness of fit statistic [20] and model discrimination was assessed using the area under the receiver operating characteristic curve [21].

**Qualitative analysis:** qualitative analysis of FGD and IDI transcripts involved coding, extracting themes and subthemes, grouping them into main categories, and supporting them with quotes from participants. The immunisation policy review examined whether government documents explicitly mention MOVs as being related to vaccination coverage and equity and preventable. The review covered the National Child Health Policy, the 5-year Health Sector Strategy: Investing in Health and Achieving the Millenium Development Goals (MDGs) 2007-2011, and Sudan's National Health Policy 2017-2030, the Sudan comprehensive multi-year plan (c-MYP) for immunisation 2012-2016 and cMYP 2021-2025 and 2023 Sudan Equity Accelerator Fund (EAF) proposal.

**Ethical considerations:** the study protocol was approved by the Ethical Review Committee of Mahidol University (COA. No. MUPH 2023-056, date 22^nd^ June 2023) and from Federal Ministry of Health (Sudan) research committee (1-2-23, 27 March 2023). Prior to each interview, caregivers, health workers, health administrators and officials were informed about the objectives of the study and that if they accepted to take part in the study, their participation was voluntary, and that they were free to withdraw at any time if they felt needed to do so. They were also informed that names of respondents will not be collected, and information will be anonymised. Accordingly, they were asked to provide verbal consent. Interviews were conducted by trained interviewers in the appropriate language.

## Results

**Figure 1:**
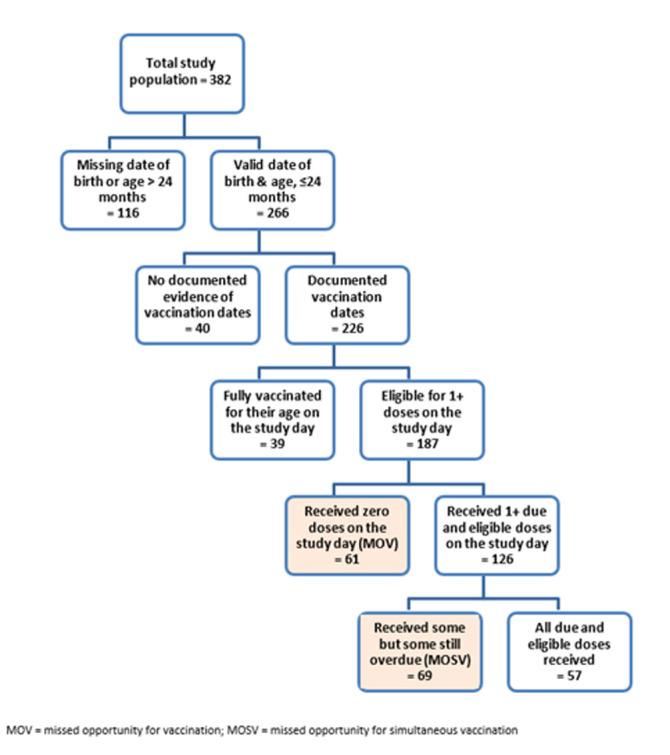
eligibility tree for identifying missed opportunities for vaccination

The study interviewed parents or caregivers of 382 children; 226 of them were under the age of 24 months and had the child's vaccination card that documented one or more vaccination dates. Characteristics of those children and caregivers are described in Table 1. The childhood vaccination schedule for Sudan at the time of the study is listed in Table 2. The number of children who were eligible for doses and their vaccination outcomes are documented in Figure 1.

**Table 1:** child and caregivers characteristics (n=226)

Category	N		%
**Child age mean=8.3 months, standard deviation=5.7 months**			
<1 year	182		80.5
1-2 years	44		19.5
**Child sex**			
Male	102		45.1
Female	124		54.9
**Place of birth**			
At home	125		54.6
At health facility	104		45.4
**Child vaccination status**			
Fully vaccinated		39	17.3
Partially vaccinated		185	81.9
Un-vaccinated		2	0.9
**Caregivers’ education**			
No formal education		31	13.7
Did not complete primary (less than 6 years)		11	4.9
Completed primary education		62	27.4
Completed secondary education		62	27.4
University education		55	24.3
Above university		5	2.2
**Caregivers’ occupation**			
Housewife		184	81.4
Employee or laborer		23	10.2
Self-employed		2	0.9
Boss or employer (own business)		0	0
Student		1	0.4
Teacher		15	6.6
Other		1	0.4
**Relationship to the child**			
Mother/Father		223	98.7
Uncle/Aunt		1	0.4
Brother/sister		1	0.4
Other		1	0.4

**Table 2:** childhood vaccination schedule for Sudan in 2023

Vaccine	Disease	Birth	6 weeks	10 weeks	14 weeks	9 months	18 months
Bacillus Calmette-Guérin	Tuberculosis	X	-	-	-	-	-
Oral Polio	Polio	X	X	X	X	-	-
Pentavalent	Diphtheria, tetanus, whooping cough (pertussis), hepatitis B, and *Haemophilus influenzae* type b	-	X	X	X	-	-
Rotavirus	Rotavirus	-	X	X	X	-	-
Pneumococcal Conjugate	Pneumococcal disease	-	X	X	X	-	-
Meningococcal Polysaccharide	Meningococcal disease	-	-	-	-	X	-
Measles	Measles	-	-	-	-	X	X
Yellow fever	Yellow fever	-	-	-	-	X	-

On the study day, 187 children were eligible for 678 doses, or an average of 3.6 doses per child. Sixty-one of those children (32.6%) did not receive any doses on the study day; those are MOVs. The remaining 126 children received 452 doses (an average of 3.6 doses per child), of which 7 were given early (they were not eligible for those) and 445 were due. Fifty-seven children (30.5% of those eligible for doses) received all the doses they were due. Sixty-nine (36.9% of those eligible for doses) received some doses but not all they were due; they experienced MOSVs. Those 69 children received an average of 3.3 doses and missed an average of 2.1 doses. So, of 187 children who were eligible for vaccination, 130 (69.5%) experienced either MOVs or MOSVs. Of the 678 doses that were due, 445 (65.6%) were received and 233 (34.3%) were not. Table 3 summarises the frequency of valid doses and MOSVs both before and on the study day by antigen. Rates of MOSVs before the study date were quite consistent with the rates of MOVs and MOSVs on the study day across all antigens.

**Table 3:** vaccination outcomes among children who were eligible for vaccine doses, before the study day (BSD) and on the study day (OSD)

	Documented Vaccination Visits before the Study Day (BSD)			Documented Vaccination Visits on the Study Day (OSD)		
Antigen	Received valid dose (%)	Experienced MOSV (%)	Total Number of Visits where Children were Eligible for the Dose (N)	Received valid dose (%)	Experienced MOV or MOSV (%)	Total Number of Children Eligible for the Dose (N)
BCG	36.5	63.5	304	36.8	63.2	114
OPV0	73.5	26.5	49	0.0	0.0	0
PENTA	85.4	14.6	288	85.7	14.3	112
PCV	82.0	18.0	305	82.1	17.9	117
OPV	95.1	4.9	283	93.5	6.5	107
ROTA	73.4	26.6	319	68.4	31.6	133
MPSV	50.0	50.0	52	56.3	43.8	64
MCV	62.3	37.7	53	62.7	37.3	59
YF	50.9	49.1	53	60.3	39.7	63

MOSV = missed opportunity for simultaneous vaccination; MOV = missed opportunity for vaccination; BCG = Bacillus Calmette-Guérin; OPV0 = birth dose of oral polio vaccine; PENTA = pentavalent vaccine; PCV = Pneumococcal Conjugate Vaccine; OPV = oral polio vaccine; ROTA = rotavirus vaccine; MPSV = Meningococcal Polysaccharide Vaccine; MCV = Measles containing vaccine; YF = yellow fever

**Association between MOVs and influencing factors:** Table 4 presents survey-adjusted univariable and multivariable logistic regression analyses of the association between whether the child experienced one or more MOVs on or before the study day (dependent outcome) and independent variables. In univariable analysis, six variables were associated with the outcome, having a p-value smaller than 0.2: age of the child, place of birth, caregiver education, caregiver occupation, vaccination decision-maker, and mode of transport to the hospital on the study day. Those variables were candidates for multivariable analysis. In the final multivariable regression, after adjusting for other variables and eliminating those with p-values above 0.05, three covariates remained. MOVs were less prevalent in a) children who were born at a health facility (aOR 0.52, 95% CI 0.29-0.95; P=.03), b) children whose caregiver has secondary or higher education (aOR 0.35, 95% CI 0.18-0.67; P=0.002), and c) families where the father alone makes decisions about childhood vaccination (aOR 0.31, 95% CI 0.14-0.69; P=0.004). The model fits the data reasonably well according to the Archer-Lemeshow goodness of fit statistic (p=0.596 with 9 groups). The area under the receiver operator characteristic curve is 0.70, which Hosmer *et al*. describe as “acceptable discrimination” [21] (Section 5.2.4).

**Table 4:** association of MOVs with independent variables

Variable	MOV		Univariate Logistic Regression			Multivariate Logistic Regression		
	Yes (n=152) n (%)	No (n=74) n (%)	Crude OR	95% CI	P-value	Adjusted OR	95% CI	P-value
**Age**								
<1 year	116 (63.7)	66 (36.3)	Reference					
1-2 years	36 (81.8)	8 (18.2)	2.56	1.10-5.92	0.03			
**Gender**								
Boys	70 (68)	33 (32.0)	Reference					
Girls	82 (66.7)	41 (33.3)	0.94	0.53-1.67	0.84			
**Place of birth**								
At home	90 (72.6)	34 (27.4)	Reference			Reference		
At health facility	62 (60.8)	40 (39.2)	0.59	0.34-1.00	0.05	0.52	0.29-0.95	0.03
**Type of hospital**								
Public	137 (67.1)	67 (32.8)	0.95	0.37-2.48	0.92			
Private	15 (68.2)	7 (31.8)	Reference					
**Caregiver education**								
Less than secondary	79 (76)	25 (24.1)	Reference			Reference		
Secondary and above	73 (59.8)	49 (40.2)	0.47	0.26-0.84	0.01	0.35	0.18-0.67	0.002
**Caregiver occupation**								
Housewife	130 (70.7)	24 (29.4)	2.19	1.11-4.29	0.02			
Others	22 (52.4)	20 (47.6)	Reference					
**Decision-making on child vaccination**								
Consensus	72 (69.2)	32 (30.8)	Reference			Reference		
Father	25 (50.0)	25 (50.0)	0.44	0.23-0.88	0.02	0.31	0.14-0.69	0.004
Mother	55 (76.4)	17 (23.6)	1.44	0.77-2.68	0.25	1.59	0.81-3.09	0.81
**Mode of transport to hospital**								
Motor transport	104 (64.6)	57 (35.4)	0.65	0.34-1.21	0.17			
Other	48 (73.9)	17 (26.2)	Reference					
**Time to go to hospital**								
<30 mins	87 (66.9)	43 (33.1)	Reference					
>30 mins	65 (67.7)	31 (32.3)	1.04	0.61-1.78	0.90			

MOV = Missed opportunity for vaccination; OR = odds ratio; CI = confidence interval; regression outcome = 1 if child experienced one or more MOVS and 0 otherwise; all explanatory factors were coded using binary indicator variables.

**Thematic analysis of interviews and focus groups:** interviews and focus group discussions yielded six themes as likely to contribute to MOVs and MOSVs. Additional details, including insightful quotes from interview participants, are in the supplementary document (Annex 1). The themes include:

***Service delivery management system:*** access barriers included vaccine shortages, notably for BCG and measles, and shortages of syringes, immunisation cards, and data reporting tools. Factors hindering vaccination included poor collaboration between hospital units, long waiting times, and crowded sites.

***Human resources and staffing:*** interviewees noted a shortage of vaccinators and the need to recruit more to open new vaccination sites. Health workers also lacked recent refresher training on immunisation and MOVs.

***Access to services:*** barriers include limited vaccination service hours, inconvenient service locations, and some long travel distances with associated travel costs.

***Infrastructure:*** specifically lack of seating for everyone who needs to wait and some inadequate ventilation in the facilities.

***Compliance and prioritization:*** non-compliance with immunisation policies, negative beliefs, ignorance about the importance of timely vaccination, and competing job duty priorities**.**

***Vaccine hesitancy and rumours:*** fear of side effects and reluctance from health workers to give multiple injections in one visit.

**Policy documents review:** among the documents and policies that were reviewed, only one - the Sudan Equity Accelerator Fund proposal which was drafted by the Federal Ministry of Health in 2023 - mentioned MOV reduction as a strategy to improve coverage and equity [22].

## Discussion

Achieving high and equitable immunisation coverage directly supports several SDGs and the WHO IA2030 goals [23]. Improving vaccination timeliness can increase coverage and equity [24]. This study, the first of its kind in Sudan and White Nile State, aimed to a) estimate the prevalence of MOVs among children ages 0-24 months in hospitals of White Nile State, b) assess factors associated with MOVs c) document stakeholders' perceptions of underlying causes of MOVs in those hospitals, and d) review policy documents for material on how to minimize MOVs.

Data analysis from a study done by Save the Children [25], shows a clear association between immunisation coverage and various health determinants, which vary across and within countries. That study and a rich set of other earlier studies documented numerous factors that can contribute to MOVs:

Stockouts of vaccines and syringes and vaccination cards [15,26].

Health worker errors. It is important to educate health workers to check ages and follow guidelines on age eligibility for valid doses [27]. Most doses given BSD and OSD were valid, but 3.2% of doses OSD and 12.6% BSD were invalid, given too early [5,28-30]. Invalid doses require revaccination after an appropriate time interval, which can be costly and burdensome for parents and puzzling for healthcare workers to record on home-based cards. The simple lack of a space to record a date of revaccination on the card may be an impediment to administering a valid dose after an invalid dose. The recommended practice is to assess children's vaccination status during any health facility visit [1,31,32].

Reluctance to vaccinate sick children [33,34].

Reluctance to administer multiple injections in one visit [24,32]. Evidence indicates that parental acceptance of multiple injections can improve if healthcare workers provide positive recommendations and assurances [35].

Refusal due to false understanding of vaccination contraindications [9,32].

Restricted clinic hours and limiting certain vaccines to specific days cause dissatisfaction among parents/caregivers [17].

Fear of adverse events following immunisation (AEFIs) affects both health workers' willingness to administer doses and parents' acceptance of vaccines [17,18].

There is also a lack of integration between curative and preventive services within hospitals. Non-immunisation staff are often not trained or in the habit of checking vaccination history, especially in outpatient and pediatric wards [36]. A similar study in Jordan found that health workers' failure to adhere to or misunderstand policies, guidelines, and procedures, including reviewing children's vaccination status during non-vaccination visits, contributes to MOVs [24]. Health workers often won't vaccinate without documented vaccination history or a card, even though children visiting these settings often don't bring cards [37,38].

A lack of refresher training and orientation to address MOV [39,40].

More MOSVs if the child is older; healthcare workers may be less likely to screen for eligibility and caregivers may be less likely to still carry older children's cards [31,41].

Living far from health services or having transport challenges [42,43].

Respondents in this study mentioned all of those reasons. Healthcare workers experienced intermittent supplies of vaccines, syringes, and other ancillary items, particularly BCG vaccines and 0.05ml syringes. Qualitative findings support this, with 18.9% of health workers interviewed in this study reporting that they lack enough vaccines and 24.3% lack other supplies. The items most often out of stock were BCG and measles vaccines, vaccination cards, and syringes.

In addition to the items listed above, White Nile State hosts many refugees and internally displaced persons, so it faces barriers due to conflict and displacement, missing documentation of earlier doses, and population registration challenges [44-46]. And as a culture, many Sudanese parents bring their newborn children for first vaccination visits at the age of 6 weeks or older, despite WHO and Sudan national guidelines recommending BCG vaccination at birth or within 7 days (Table 2) [30]. Consequently, children not born at facilities offering BCG vaccination are at high risk of missing timely doses. More than half the children in this study were born at home. That risk may be compounded by health workers' reluctance to open a 20-dose BCG vial or a 10-dose measles vial for a single child, because of guidance to not waste large portions of the vaccine in a vial. BCG and measles vaccinations are often scheduled on specific days to minimize vaccine wastage, but those days may not be convenient for caregivers and can lead to crowded clinics with long waits, which may, in turn, result in dissatisfaction with health services [39,47] and even later doses for children whose parents decide not to endure a long, possibly uncomfortable wait at the clinic.

This study has some strengths. It captured data from most of the hospitals that provide immunisation services in White Nile State. The MISS VCQI software uses a consistent set of computational rules and criteria to document MSVs and MOVs. It calculates MOV rates for all of the child's documented vaccination visits, both on and before the study day, giving a thorough assessment of the child's immunisation experiences. A limitation of the study is its analytic focus solely on children with documented vaccination records (cards) who brought them to the hospital. It missed checking vaccination status from children whose caregivers did not have the card in their possession at the time of the exit interview. Another factor is that the study was conducted in White Nile State after the war in Khartoum, with many displaced families. Many visitors to hospitals may not have had vaccination cards or proof of vaccination history due to recent sudden displacement. Finally, logistic regression analysis is based on an assumption of a rigorously random probability sample, which does not hold here, so those results should be considered indicative of meaningful factors and correlations but perhaps not unbiased.

## Conclusion

Sudan has persistent vaccination challenges. It would be helpful for the FMoH to develop MOV guidance to introduce into national and state level policy documents. Vaccine and syringe and card supply infrastructure should be strengthened. It could be instructive to develop and deliver MOV refresher training for healthcare workers. Screening and immunising children at every health contact should be institutionalized, especially in clinical departments, and especially for older children [43,48]. Vaccination awareness could be raised among caregivers via attractive communication campaigns describing benefits of timely vaccination and putting to rest false ideas about contraindications [49]. The second year of life immunisation platform could be strengthened with a focus on administering previously missed doses. And MOV assessments could be conducted periodically to track progress and address causes that persist or evolve.

### What is known about this topic

- Missed Opportunities for Vaccination (MOV) contribute to low and inequitable vaccination coverage;
- MOVs may be more preventable and their causes more addressable than some other barriers to vaccination;
- Beyond improving immunisation coverage, the aim of reducing MOVs is to promote synergy between programmes and improve health service delivery.

### What this study adds

- This work quantifies the prevalence of MOVs at public and private hospitals in White Nile State, Sudan and fills a knowledge gap in this area;
- It identifies likely causes of MOVs and evaluates whether current policies and guidelines are adequate to resolve the issue;
- The study's findings will help health authorities and partners refine strategies and resources to address MOVs and improve vaccination coverage and equity.

## Annex

**Annex 1**: supplementary material for MOV study White Nile State (Sudan)_2023 (PDF-116KB)

## References

[1] World Health Organization (WHO) Immunization Agenda 2030: A Global Strategy To Leave No One Behind.

[2] World Health Organization (WHO) The private sector, universal health coverage and primary health care, technical series on primary health care.

[3] Chopra M, Weis J (2018). Closing equity gaps in immunisation: Relevance of human rights-based and behavioural economics approaches.

[4] World Health Organization WHO/UNICEF estimates of national immunization coverage.

[5] World Health Organization (WHO) Methodology for the assessment of missed opportunities for vaccination.

[6] Jacob N, Coetzee D (2015). Missed opportunities for immunisation in health facilities in Cape Town, South Africa. S Afr Med J.

[7] Rhoda DA, Prier ML, Clary CB, Trimner MK, Velandia-Gonzalez M, Danovaro-Holliday MC (2021). Using Household Surveys to Assess Missed Opportunities for Simultaneous Vaccination: Longitudinal Examples from Colombia and Nigeria. Vaccines.

[8] Borras-Bermejo B, Panunzi I, Bachy C, Gil-Cuesta J (2022). Missed opportunities for vaccination (MOV) in children up to 5 years old in 19 Médecins Sans Frontières-supported health facilities: a cross-sectional survey in six low-resource countries. BMJ Open.

[9] Sridhar S, Maleq N, Guillermet E, Colombini A, Gessner BD (2014). A systematic literature review of missed opportunities for immunization in low-and middle-income countries. Vaccine.

[10] Tamuzi JL, Katoto PDMC, Ndwandwe DE, Wiysonge CS, Nyasulu PS (2025). Prevalence of missed opportunities for vaccination (MOV) indicators among children aged 12-23 months in sub-Saharan African countries: An individual-level meta-analysis of DHS and MICS national household data surveys. Hum Vaccin Immunother.

[11] Federal Ministry of Health Sudan's National Health Policy 2017-2030.

[12] Pan American Health Organization Methodology for the Evaluation of Missed Opportunities for Vaccination.

[13] Creswell JW, Clark VL (2017). Designing and conducting mixed methods research. Sage Publications.

[14] Georgia State University Research Guides - Mixed Methods.

[15] World Health Organization (WHO) Planning Guide to Reduce Missed Opportunities for Vaccination.

[16] Ogbuanu IU, Li AJ, Anya BM, Tamadji M, Chirwa G, Chiwaya KW (2019). Can vaccination coverage be improved by reducing missed opportunities for vaccination? Findings from assessments in Chad and Malawi using the new WHO methodology. PLoS One.

[17] Kaboré L, Meda B, Médah I, Shendale S, Nic Lochlainn L, Sanderson C (2020). Assessment of missed opportunities for vaccination (MOV) in Burkina Faso using the World Health Organization's revised MOV strategy: Findings and strategic considerations to improve routine childhood immunization coverage. Vaccine.

[18] Li AJ, Peiris TSR, Sanderson C, Nic Lochlainn L, Mausiry M, da Silva RBJBM (2019). Opportunities to improve vaccination coverage in a country with a fledgling health system: Findings from an assessment of missed opportunities for vaccination among health center attendees-Timor Leste, 2016. Vaccine.

[19] StataCorp The complete statistical software for data science.

[20] Archer KJ, Lemeshow S (2006). Goodness-of-fit test for a logistic regression model fitted using survey sample data. The Stata Journal.

[21] Hosmer DW, Lemeshow S, Sturdivant RX Applied Logistic Regression.

[22] Sudan Federal Ministry of Health (2023). Supporting Narrative for Theory of Change for Gavi Equity Acceleration Fund.

[23] Decouttere C, De Boeck K, Vandaele N (2021). Advancing sustainable development goals through immunization: a literature review. Global Health.

[24] Missed Opportunities for Vaccination MISSED OPPORTUNITIES FOR VACCINATION ASSESSMENT REPORT: findings, lessons learned and experiences from a high-performing middle-income country.

[25] Save the Children Finding the FINAL FIFTH: Inequalities in immunisation.

[26] Olorunsaiye CZ, Langhamer MS, Wallace AS, Watkins ML (2017). Missed opportunities and barriers for vaccination: a descriptive analysis of private and public health facilities in four African countries. Pan Afr Med J.

[27] Lee D, Lavayen MC, Kim TT, Legins K, Seidel M (2023). Association of vaccine stockout with immunisation coverage in low-income and middle-income countries: a retrospective cohort study. BMJ Open.

[28] Abt Associates (2018). Role of Private Sector Providers in Malawi National Immunisation Programme.

[29] UNICEF Regional Office for Middle East and North Africa (MENARO) (2021). Country Profiles for Immunization 2020.

[30] World Health Organization (WHO) WHO recommendations for routine immunization - summary tables.

[31] Loevinsohn BP, Gareaballah E (1992). Missed opportunities for immunization during visits for curative care: a randomized cross-over trial in Sudan. Bull World Health Organ.

[32] Hutchins SS, Kim-Farley RJ (1993). Studies of missed opportunities for immunization in developing and industrialized countries. Bull World Health Organ.

[33] Holt E, Guyer B, Hughart N, Keane V, Vivier P, Ross A (1996). The contribution of missed opportunities to childhood underimmunization in Baltimore. Pediatrics.

[34] McConnochie KM, Roghmann KJ (1992). Immunization opportunities missed among urban poor children. Pediatrics.

[35] Levin A, Kaddar M (2011). Role of the private sector in the provision of immunization services in low-and middle-income countries. Health Policy Plan.

[36] Dolan SB, Patel M, Hampton LM, Burnett E, Ehlman DC, Garon J (2017). Administering Multiple Injectable Vaccines During a Single Visit-Summary of Findings From the Accelerated Introduction of Inactivated Polio Vaccine Globally. J Infect Dis.

[37] Fatiregun AA, Lochlainn LN, Kaboré L, Dosumu M, Isere E, Olaoye I (2021). Missed opportunities for vaccination among children aged 0-23 months visiting health facilities in a southwest State of Nigeria, December 2019. PLoS One.

[38] World Health Organization (2019). Intervention guidebook for implementing and monitoring activities to reduce missed opportunities for vaccination.

[39] Li AJ, Tabu C, Shendale S, Okoth PO, Sergon K, Maree E (2020). Qualitative insights into reasons for missed opportunities for vaccination in Kenyan health facilities. PLoS One.

[40] Szilagyi PG, Rodewald LE (1996). Missed opportunities for immunizations: a review of the evidence. J Public Health Manag Pract.

[41] Mohamed Hayir TM, Magan M, Mohamed L, Mohamud M, Muse A (2020). Barriers for full immunization coverage among under 5 years children in Mogadishu, Somalia. J Family Med Prim Care.

[42] Jani JV, De Schacht C, Jani IV, Bjune G (2008). Risk factors for incomplete vaccination and missed opportunity for immunization in rural Mozambique. BMC Public Health.

[43] Nnaji CA, Wiysonge CS, Adamu AA, Lesosky M, Mahomed H, Ndwandwe D (2022). Missed Opportunities for Vaccination and Associated Factors among Children Attending Primary Health Care Facilities in Cape Town, South Africa: A Pre-Intervention Multilevel Analysis. Vaccines.

[44] UNICEF State Profile-White Nile.

[45] United Nations Children's Fund (UNICEF) The UNICEF Immunization Roadmap 2018-2030.

[46] World Health Organization (WHO) Inequality monitoring in immunization: a step-by-step manual.

[47] World Health Organization (WHO) WHO Policy Statement: Multi-dose Vial Policy (MDVP).

[48] Hwang A, Veira C, Malvolti S, Cherian T, MacDonald N, Steffen C (2020). Global Vaccine Action Plan Lessons Learned II: Stakeholder Perspectives. Vaccine.

[49] Albaugh N, Mathew J, Choudhary R, Sitaraman S, Tomar A, Bajwa IK (2021). Determining the burden of missed opportunities for vaccination among children admitted in healthcare facilities in India: a cross-sectional study. BMJ Open.

